# A Molecular Dynamics Study of Single-Gas and Mixed-Gas N_2_ and CH_4_ Transport in Triptycene-Based Polyimide Membranes

**DOI:** 10.3390/polym15183811

**Published:** 2023-09-18

**Authors:** Ioannis Tanis, David Brown, Sylvie Neyertz, Milind Vaidya, Jean-Pierre Ballaguet, Sebastien Duval, Ahmad Bahamdan

**Affiliations:** 1 Univ. Savoie Mont Blanc, Univ. Grenoble Alpes, CNRS, Grenoble INP, LEPMI, 38000 Grenoble, France; david.brown@univ-smb.fr (D.B.); sylvie.neyertz@univ-smb.fr (S.N.); 2 Saudi Aramco, Research & Development Center, P.O. Box 62, Dhahran 31311, Saudi Arabia; milind.vaidya@aramco.com (M.V.); peterball128@yahoo.fr (J.-P.B.); sebastien.duval@aramco.com (S.D.); ahmad.bahamdan@aramco.com (A.B.)

**Keywords:** molecular dynamics, gas separation, triptycene, polyimide membranes

## Abstract

Fluorinated polyimides incorporated with triptycene units have gained growing attention over the last decade since they present potentially interesting selectivities and a higher free volume with respect to their triptycene-free counterparts. This work examines the transport of single-gas and mixed-gas N_2_ and CH_4_ in the triptycene-based 6FDA-BAPT homopolyimide and in a block 15,000 g mol^−1^/15,000 g mol^−1^ 6FDA-mPDA/BAPT copolyimide by using molecular dynamics (MD) simulations. The void-space analyses reveal that, while the free volume consists of small-to-medium holes in the 6FDA-BAPT homopolyimide, there are more medium-to-large holes in the 6FDA-mPDA/BAPT copolyimide. The single-gas sorption isotherms for N_2_ and CH_4_ over the 0–70 bar range at 338.5 K show that both gases are more soluble in the block copolyimide, with a higher affinity for methane. CH_4_ favours sites with the most favourable energetic interactions, while N_2_ probes more sites in the matrices. The volume swellings remain limited since neither N_2_ nor CH_4_ plasticise penetrants. The transport of a binary-gas 2:1 CH_4_/N_2_ mixture is also examined in both polyimides under operating conditions similar to those used in current natural gas processing, i.e., at 65.5 bar and 338.5 K. In the mixed-gas simulations, the solubility selectivities in favour of CH_4_ are enhanced similarly in both matrices. Although diffusion is higher in 6FDA-BAPT/6FDA-mPDA, the diffusion selectivities are also close. Both triptycene-based polyimides under study favour, to a similar extent, the transport of methane over that of nitrogen under the conditions studied.

## 1. Introduction

The separation of small gas molecules is of major importance for the recovery of valuable gases and fuel production [1,2,3], as well as in other industrial processes, such as air separation, food packaging, and pollution control [2,4,5,6]. Polymeric-membrane-mediated gas separation appears as one of the preferred choices for the gas separation industry over traditional processes, such as cryogenic distillation, filtration, ion exchange, and thermal and pressure-swing adsorption, due to its simpler fabrication, lower costs, energy efficiency, and reduced environmental impact [7,8,9,10,11,12,13,14,15]. Recent studies report a class of hydrocarbon polymers that can achieve both high selectivity and high gas permeability in membrane separations for many gas mixtures [16], whereas other current works highlight the contribution of machine learning models to the optimisation of membrane fabrication and design [17,18,19,20,21,22,23].

Applications of polymer membranes in natural gas processing often involve denitrogenation, i.e., the removal of excess nitrogen from natural gas reservoirs, as the former lowers the thermal efficiency (heating value) of natural gas and increases transport volumes. In addition, most pipeline standards require that natural gas contains less than 4% N_2_ [24,25,26]. In general, nitrogen is difficult to separate from methane due to their similar kinetic diameters (3.64 and 3.8 Å, respectively) [27] and physical properties. However, membrane-mediated separation is accomplished via differences in both gas solubilities and diffusivities [28,29,30].

The transport of gases through dense membranes is a solution–diffusion process, where gas molecules are initially sorbed into the membrane surface, diffuse across the membrane via a series of jumps through inter-connected cavities, and, finally, desorb at the downstream compartment of the membrane [31,32]. The ability of a membrane to separate a given gas pair is usually assessed by its permeability and its selectivity or separation factor. In the case where the downstream pressure is negligible compared to the upstream pressure of the gas feed, the permeability, *P*, is usually expressed as the product of the solubility and diffusion coefficients (Equation (1)):

(1) $$P_{i}=S_{c_{i}}D_{i}$$

The diffusion coefficient, $D_{i}$, is the kinetic contribution to the permeability, whereas the solubility coefficient, $S_{c_{i}}$, characterises the partitioning of the gas penetrants into the polymer and is given by the ratio of the concentration of the gas in the polymer, $C_{i}$, to the partial pressure, $p_{i}$, of the gas to which it is exposed (Equation (2)):

(2) $$S_{c_{i}}=\frac{C_{i}}{p_{i}}$$

Assuming that a gas, A, is more permeable than a gas, B, the separation capability of a membrane for a given gas pair, α*_A/B_*, is given by the ratio of the permeabilities (Equation (3)):

(3) $$\alpha_{A/B}=\frac{P_{A}}{P_{B}}=\left(\frac{D_{A}}{D_{B}}\right)\left(\frac{S_{A}}{S_{B}}\right)$$

Amongst the various polymer families used in gas separations, aromatic polyimides (PIs) have attracted special attention due to their satisfactory chemical and thermal stabilities, as well as their resistance to mechanical stresses [7,33,34,35]. Moreover, hexafluorodianhydride (6FDA)-based PIs appear as particularly appealing candidates in separation processes as they provide considerably higher selectivities than other dianhydrides [36,37,38].

Despite the exceptional properties of aromatic PIs, there still exist several challenges for long-term industrial applications, which are primarily related to the permeability/selectivity tradeoff, physical ageing, and membrane plasticisation in the presence of highly condensable gases [39]. These issues can partly be circumvented at the macromolecular level by integrating bulky moieties and/or rigid tortuous elements in the polymer backbone in order to inhibit polymer chain packing and increase the fractional free volume (FFV) of the material [39,40].

Recent studies have highlighted the usage of iptycene molecules as building elements to address some of the challenges mentioned earlier [7,41,42,43,44]. Triptycene is a rigid three-dimensional molecular unit with three blades, each composed of a benzene ring [45,46]. Several groups have synthesised novel triptycene-based PIs and examined structure–property relationships that can guide the rational design of improved-performance membranes targeted for gas separations. One of the first-synthesised triptycene-based PIs bears the commercial name 6FDA-DATRI [47]. This PI was found to have a higher FFV than other 6FDA-based PIs and a CO_2_/CH_4_ selectivity that outperforms any commercial PI. In addition, 6FDA-DATRI presents a satisfactory resistance to plasticisation, which was attributed to the π–π interactions of the triptycene benzene blades perpendicular to the polymer backbone [47]. Wiegand et al. synthesised a 6FDA-1,4-triptycene polyimide, also known as 6FDA-BAPT, and compared its gas separation performance with other polyimides [40]. They measured an ideal selectivity of 1.78 for the N_2_/CH_4_ pair at *T* = 35° and *p* = 9 bar and observed a favourable free-volume size distribution induced by the symmetric nature of the triptycene unit [40]. Weidman and co-workers examined the physical ageing properties of 6FDA-BABT and found that the addition of substituent groups resulted in an improved permeability over time due to steric hindrance effects [48]. Other groups examined the permselectivity properties of thermally rearranged triptycene-containing polybenzoxazoles (PBO) derived from polyimide precursors with triptycene units [49,50]. Specifically, triptycene-containing TR-PBO membranes synthesised by Luo et al. were found to have an enhanced N_2_/CH_4_ ideal selectivity greater than 2 at *p* = 11 atm and *T* = 35 °C [49].

As a complement to experiments, molecular dynamics (MD) simulations are a useful tool to study structure–property relationships and the gas separation performance of triptycene-based PIs. In particular, Cho et al. [47] examined the free-volume distribution and gas transport in 6FDA-DATRI membranes in a combined experimental and simulation study. Their calculations yielded an FFV higher than other polyimide membranes, whereas a selectivity of 1.3 for N_2_/CH_4_ at *p* = 1 bar and *T* = 35 °C was reported [47]. A combination of MD, Monte Carlo (MC), and ab initio simulations was used by Chen et al. [51] to examine the structural properties and transport behaviour of a triptycene-based polymer with intrinsic microporosity (PIM) membranes. They showed that the incorporation of the triptycene moiety rigid structure disrupts the polymer chain packing, leading to enhanced diffusion selectivities for the O_2_/N_2_ pair [51]. In an analogous study of Ghasemnejad-Afshar et al., it was shown that PIM membranes with larger side branch groups in their polymeric chain structure are more rigid, which is due to restriction in chain packing and cavity formation between polymer chains [52]. More recently, Balcik et al. [53] investigated the pure- and mixed-gas CO_2_/CH_4_ separation properties of a triptycene–Tröger’s base ladder PIM-Trip-TB polymer. They found that the CO_2_/CH_4_ solubility selectivity is enhanced under mixed-gas conditions, but despite the intra-chain rigidity, an increase in FFV upon exposure to plasticising CO_2_ leads to a loss in diffusion solubility [53].

**Figure 1:**
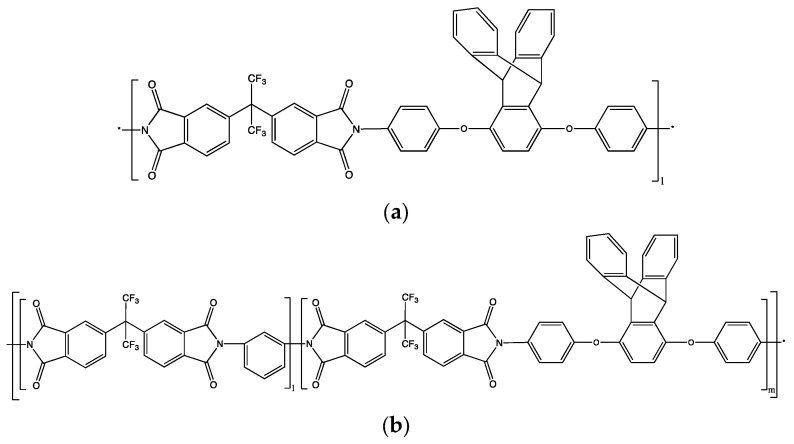
Schematic representation of the chemical structures of (**a**) the 6FDA-BAPT homopolyimide with *l* = 45 monomers and (**b**) the block 6FDA-mPDA/BAPT copolyimide with *l* = 29 monomers of 6FDA-mPDA followed by *m* = 17 monomers of 6FDA-BAPT.

The aim of this contribution is to examine the transport of the less plasticising N_2_ and CH_4_ in triptycene-based homo- and block copolyimides using MD simulations. In particular, we assess the free-volume distribution and transport behaviour of 6FDA-1,4-triptycene, i.e., 6FDA-BAPT, for both single-gas and mixed-gas feeds. Given its promising transport properties [40], it appears interesting to combine it with the 6FDA-mPDA polyimide, which exhibits better packing on its own and is selective over nitrogen [54,55]. To this end, we also examine the performance of a block copolyimide made of the aforementioned base homopolyimides. The chemical formulae of the polyimides under study are shown in Figure 1.

As shown in previous work, an estimation of the permselectivity behaviour of the polyimides exclusively from single-gas conditions can lead to misleading findings [56,57,58,59]. Therefore, permeation studies were carried out for pure N_2_ and CH_4_ loadings and also for binary mixtures at a ratio equivalent to those found in some natural gas reservoirs, i.e., as used before [59], a 2:1 mixture of CH_4_/N_2_ at 65.5 bar and at 338.5 K. These are the processing conditions that were specified by the industrial partner. The preparation of the amorphous polyimide models, along with the examination of their void space, is described in Section 2. Section 3 presents the simulation protocols and the methods to measure model gas permeation in the polyimide matrices for both pure- and mixed-gas feeds at 338.5 K. The compared single-gas and binary-gas analyses of N_2_ and CH_4_ transport in the 6FDA/BAPT polyimide and its 6FDA-mPDA/BAPT copolyimide are presented in Section 4.

## 2. Modelling the Pure Triptycene-Based 6FDA-Polyimides

### 2.1. Preparation of the Amorphous Polyimide and Copolyimide Models

All simulations were carried out with the aid of the scalar and parallel versions of the general-purpose *gmq* software version VI [60]. As the transport characteristics of a membrane are governed by the core rather than by the membrane–gas interface, the triptycene-based polyimide representations were bulk models in three dimensions with periodic boundary conditions [61]. The fully atomistic amorphous models of 6FDA-BAPT and 6FDA-mPDA/BAPT contained each 6 chains and were prepared via the hybrid pivot Monte Carlo–molecular dynamics (PMC-MD) method [62,63]. This allows for the generation of a polymer sample at the desired density with the polymer chains bearing conformations that correspond to the equilibrium melt at the required temperature. The underlying theory stems from Flory’s hypothesis [64], according to which the configurations of polymer chains in the pure melt are determined by only a certain number of near-neighbour intramolecular interactions. The method has been thoroughly described elsewhere and validated against various polymer families [62,63]. Earlier studies on polyimide systems reported that the chain characteristic ratio, $R^{2}/n_{monomers}$, i.e., the ratio of the mean square end-to-end distance of the chains <*R*^2^> over the number of monomers, becomes number-independent from *n_monomers_* ~30 [38,39]. On these grounds, the mean molecular weight of the 6FDA-BAPT chains was ~40,000 g mol^−1^, with each chain comprising *l* = 45 monomers (Figure 1), which is consistent with experimental studies [40]. Motivated by a previous study demonstrating that block copolyimides of a similar block size show promising gas transport properties [54], we considered a system comprising two types of chains. One chain of type ABA bearing the simplified sequence (mPDA-6FDA)_30_-(BAPT-6FDA)_17_-(mPDA-6FDA)_29_ resulting in 4323 atoms per chain and another chain of type BAB with the sequence (BAPT-6FDA)_18_-(mPDA-6FDA)_29_-(BAPT-6FDA)_17_ corresponding to a total number of 4551 atoms. The number of monomers of each block is chosen as such in order to obtain a mean molar mass ratio of 15,000/15,000/150,000. A schematic representation of a chain sequence comprising *l* 6FDA-mPDA blocks and *m* 6FDA-BAPT blocks is presented in Figure 1. For both systems, the required number of uncorrelated chains was generated via PMC-MD at a temperature just above their glass transition temperature. Specifically, as the experimental measurements of the 6FDA-BAPT and 6FDA-mPDA *T*_g_ yield values close to 600 K [40,54,55], the generation temperature was set to 700 K for both polyimide systems. The melts generated by PMC-MD were, thus, obtained at a temperature where the initial configurations should be close to those of the lower-temperature glass [65].

All bond lengths were rigidly constrained to standard values via the SHAKE algorithm in order to ensure an equipartition of the kinetic energy [66]. The simulation time step was set to 1 fs. Polymer chain intra- and intermolecular interactions were parametrised via the TRIPOS 5.2 force field [67], and Lorentz–Berthelot combining rules were applied to all unlike-atom Lennard-Jones (LJ) interactions. Partial charges on the atoms (provided in the Supplementary Materials) were calculated using the *Gaussian* code [68] at the B3LYP/6-31G** level on representative three-fragment structures of the polyimides under study. Electrostatic interactions were evaluated via an optimised Ewald summation [69]. Dispersive interactions were evaluated for pairs of atoms at distances up to 10 Å for all systems, whereas the energy and pressure beyond the cut-off radius were evaluated by applying standard long-range corrections. The systems generated at 700 K were run for 500 ps with constant-volume constant-temperature dynamics (*NVT* conditions) in order to allow for local relaxations. A gradual cooling to room temperature (298 K) was performed afterwards, at a rate of −0.5 K/ps. A short relaxation run at constant volume and constant temperature (*NVT*) followed, and afterwards, the systems were switched to isobaric–isothermal (*N**P**T*) conditions. The temperature was fixed at 298 K by loose coupling to a thermostat with a coupling constant of 0.1 ps [70]. The simulation cells were allowed to relax at 1 bar by loose coupling to a barostat using a coupling constant of 5 ps [71]. Production runs were conducted for 4 ns and configurations were saved every 10 ps.

### 2.2. Densities and Void-Space Analyses of the Pure Polyimide Models

Model densities were evaluated by analysing the last 2 ns of the production trajectories at 298 K. The average densities were found to be 1.306 g cm^−3^ for 6FDA-BAPT and 1.378 g cm^−3^ for 6FDA-mPDA/BAPT with error bars being of the order of O(10^−3^). Wiegand et al. [40] measured an average density of 1.323 g cm^−3^ for 6FDA-BAPT chains of M_w_ = 41,800 g mol^−1^ at 308 K, which is in good agreement with our calculations. No experimental density measurements are available for 6FDA-mPDA/BAPT, but considering the densities of the base polyimides (~1.42 g cm^−3^ for 6FDA-mPDA [72] and ~1.32 g cm^−3^ for 6FDA-BAPT [73]) and the fact that the block copolyimide chains are of a 50:50 molar mass ratio, an average model density of ~1.38 g cm^−3^ is reasonable.

**Figure 2:**
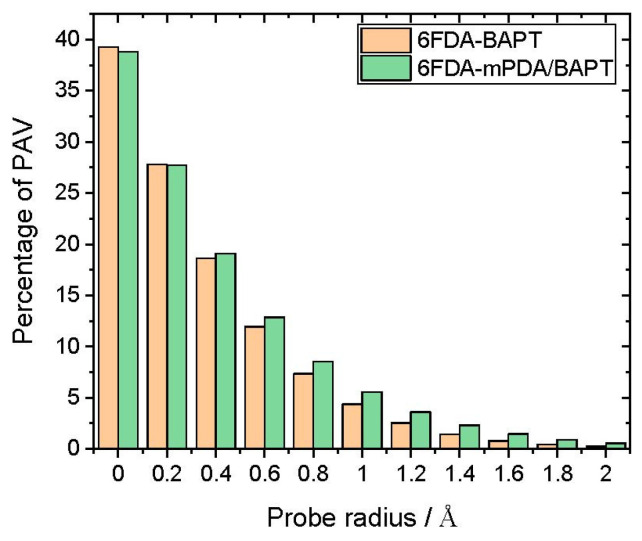
The mean percentage of probe-accessible volume (%*PAV*) as a function of the probe radius used in the trial insertions.

The polyimide void space was first analysed with the aid of a geometric technique similar to the “phantom sphere approach” that allows the determination of the volume accessible to a ghost spherical probe molecule of a preset radius inserted repeatedly into the stored polymer configurations [74,75]. We refer to our approach as the “probe-accessible volume” (*PAV*) [60]. The insertions of the spherical probes are performed independently, i.e., the current ghost probe does not interact with the other probes. An insertion is considered as “accepted” when the centre of the probe does not overlap with the polymer atoms. The latter is represented by hard spheres with standard van der Waals radii [76,77]. An illustration of the percentage of probe-accessible volume, %*PAV*, as a function of the probe size, is given in Figure 2 for both pure polymer models under study.

As expected, the %*PAV* drops systematically with the probe size (Figure 2). For small probe radii (<0.4 Å), the 6FDA-BAPT appears to contain higher %*PAV*, suggesting that it has more small holes than the copolyimide. The trend changes for larger probe radii, as 6FDA-mPDA/BAPT appears to have more accessible space than the homopolyimide. Its 6FDA-mPDA block with a molecular weight of 15,000 g mol^−1^ is probably not long enough to induce the more selective behaviour of its homopolyimide, and indeed, it further disrupts the 6FDA-BAPT block. As such, adding the 6FDA-mPDA block leads to larger-size holes, which are likely to induce even more free volume for gas transport in the copolyimide.

**Figure 3:**
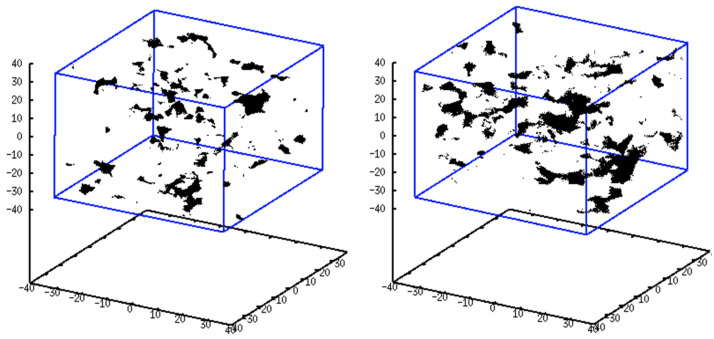
Visual characterisation of the free-volume distribution in the 6FDA-BAPT homopolyimide (**left**) and in the 6FDA-mPDA/BAPT copolyimide (**right**) obtained after performing trial insertions with a probe of radius 1.9 Å.

To obtain a more visual perception, an illustration of the free-volume distribution for each system is provided in Figure 3. The probe size radius is 1.9 Å, i.e., its diameter is close to the kinetic diameter of both N_2_ and CH_4_. This reveals that the 6FDA-BAPT homopolyimide (left picture) is indeed characterised by a rather heterogeneous distribution of accessible volume, the major part of which manifests itself in the form of small-to-medium hole sizes. The coexistence of the mPDA and BAPT diamines in the 6FDA-mPDA/BAPT copolyimide (right picture) increases the accessible space, which, in this case, is dominated by medium-to-large holes. This is consistent with the trends detected in Figure 2 for probe radii greater than 0.4 Å.

## 3. Modelling Gas Sorption and Diffusion in the Polyimide Matrices

### 3.1. MD Simulations of N_2_ and CH_4_ in the Pure-Gas Phase

Simulations of gas transport were all carried out at 338.5 K since this is a typical temperature for gas wells. As for the chemical bonds in the polymer chains, gas molecules were modelled as rigid to ensure the equipartition of kinetic energy. The parameters for the N_2_ diatomic molecule were obtained from the symmetrical two-centre Lennard-Jones plus point quadrupole pair potential (2CLJQ) from Vrabec et al. [78]. For CH_4_, a rigid 5-site model was used, with the potential parameters and partial charges being taken from the model of Yin and Mackerell [79], which has successfully been used in analogous studies [31,58]. Both the N_2_ and the CH_4_ force fields used here satisfactorily describe the experimental boiling points and self-diffusion coefficients [56].

To extract the penetrant sorption isotherms in the polymer matrices, single-gas simulations need to be conducted in the pure phase at the temperature and pressures of interest, i.e., a 0–80 bar range. For this reason, a series of *NVT* MD simulations were performed for 4 ns on systems comprising 512 CH_4_ and 1000 N_2_ molecules respectively, using box sizes calculated from experimental data in the NIST database [80].

The last 2 ns of these simulations were used for post-analyses with the configurations stored at intervals of 10 ps. After verifying that the gas systems had reached equilibrium, the gas average pressures, *p*, were extracted directly from the MD simulations. Solubilities were calculated from the standard Widom test particle insertion (TPI) method [81,82]. In order to obtain the concentrations *C_gas_*(*p*) and the corresponding solubilities in the gas phase *S_gas_*(*p*) at any pressure up to 80 bar, the data were fitted to analytical functions having the correct limiting ideal gas behaviour at low pressures. For the binary-gas mixture, the same analyses were carried out on a 2:1 CH_4_/N_2_ system with 512 CH_4_ and 256 N_2_ molecules, albeit for one set of temperature and pressure only (*T* = 338.5 K and *p* = 65.5 bar).

### 3.2. Single-Gas Uptakes in the Polyimides

In permeation experiments, the number of gas molecules sorbed in the polymer matrix depends on the external pressure, *p*, of the gas reservoir in contact with the matrix and on the temperature, *T*. In this work, an approach that iterates the pressure and accounts for the matrix changes during sorption was used to calculate the single-gas sorption isotherms for N_2_ and CH_4_ in both 6FDA-BAPT and 6FDA-mPDA/BAPT matrices [61]. The details are given elsewhere [35,62], so only the basic concepts will be presented here.

When gas molecules in the pure-gas phase are in equilibrium with the gas sorbed into the matrix, the chemical potential for the gas in the gas phase,$\;\mu_{\mathrm{gas}}$, needs to be equal to the chemical potential for the gas in the polymer phase, $\mu_{\mathrm{pol}}$. For rigid molecules, this equilibrium condition implies that the difference between the excess chemical potential, $\mu_{\mathrm{ex}}$, for the gas in its gas phase, $\mu_{\mathrm{ex}}^{\mathrm{gas}}$, and in its polymer phase, $\mu_{\mathrm{ex}}^{\mathrm{pol}}$, is related to the ratio of their concentrations in both phases, with *k_B_* being Boltzmann’s constant (Equation (4)) [83]:

(4) $$\Delta \mu_{\mathrm{ex}}=\mu_{\mathrm{ex}}^{\mathrm{pol}}-\mu_{\mathrm{ex}}^{\mathrm{gas}}=k_{B}T\;\ln\frac{C_{\mathrm{gas}}}{C_{\mathrm{pol}}}$$

In statistical mechanics, $\mu_{ex}$ is given by Equation (5) [60]:

(5) $$\;\mu_{\mathrm{ex}}=-k_{B}T\;\ln\frac{\left\langle V\exp\left(-\frac{\Delta \Phi }{k_{B}T}\right)\right\rangle }{\left\langle V\right\rangle }$$

where ΔΦ is the change in potential energy estimated using the TPI test particle insertion method, in which a penetrant molecule is virtually and repeatedly introduced into stored configurations of the corresponding model. In dense polymer systems, many millions of particle insertions have to be made in order to reach converged average values for $\;\mu_{\mathrm{ex}}$. From Equation (5), an expression for the (dimensionless) solubility is obtained (Equation (6)):

(6) $$S=\exp\left(-\frac{\mu_{\mathrm{ex}}}{k_{B}T}\right)=\frac{\left\langle V\exp\left(-\frac{\Delta \Phi }{k_{B}T}\right)\right\rangle }{\left\langle V\right\rangle }≃\langle \exp\left(-\frac{\Delta \Phi }{k_{B}T}\right)\rangle$$

The approximation in Equation (6) holds for fairly incompressible materials such as glassy polymers where volume fluctuations are minor.

As TPI methods are inefficient at high densities due to the low probability of favourable insertions, it was combined here with the excluded-volume map sampling (EVMS) approach [84,85,86], which accelerates the calculations by pre-eliminating the regions of very low insertion probabilities. This is referred to as the EVMS-TPI method. For the solubilities of the gas in the gas phases (Section 3.1), the conventional TPI is sufficient.

Equations (4)–(6) can be combined to give a convenient relationship between the ratios of the concentrations and solubilities in the two phases (Equation (7)):

(7) $$\frac{C_{\mathrm{gas}}}{C_{\mathrm{pol}}}=\exp\left(\frac{\Delta \mu_{\mathrm{ex}}}{k_{B}T}\right)=\frac{\exp\left(\frac{-\mu_{\mathrm{ex}}^{\mathrm{gas}}}{k_{B}T}\right)}{\exp\left(\frac{-\mu_{\mathrm{ex}}^{\mathrm{pol}}}{k_{B}T}\right)}=\frac{S_{\mathrm{gas}}}{S_{\mathrm{pol}}}\;\;or\;\;\;\frac{C_{\mathrm{pol}}}{C_{\mathrm{gas}}}=\frac{S_{\mathrm{pol}}}{S_{\mathrm{gas}}}$$

Equation (7) is used to determine the single-gas sorption isotherms for both N_2_ and CH_4_. For the gas phases, the gas concentration, C_gas_(*p*), and solubility, *S*_gas_(*p*), are obtained for pressures, *p*, up to ~80 bar at 338.5 K, as described in Section 3.1. For the polymer phases, both models were initially heated to 338.5 K at a rate of 0.5 K/ps. Then, based on the infinite dilution solubility coupled with *C*_gas_(*p*) and *S*_gas_(*p*) over a small pressure interval, an initial guess for the number of gas molecules to insert into a given polymer at an initial guess pressure, *p*_1_, was made. This was found to be ~50 molecules for N_2_ and ~20 molecules for CH_4_. Once inserted with EVMS-TPI, a short *NVT* 100 ps equilibration run then follows before switching to *N**P**T* conditions for a production run of at least 2 ns, the last 1 ns of which is used for post-processing. The average number density, *C*_pol_(*p*_1_), and solubility, *S*_po*l*_(*p*_1_), of the gas in the polymer phase are determined at pressure *p*_1_. Following this, the quantities *C*_pol_(*p*_1_)*/C*_gas_(*p*) and *S*_pol_(*p*_1_)*/S*_gas_(*p*) are plotted separately as a function of the pressure, *p*, of the gas phase. The point of intersection of the two curves gives the second approximation, *p*_2_. Then, another simulation of the gas in the polymer phase is carried out at *p*_2_, which provides a third estimate, and so on. The maximum number of iterations to reach convergence at 338.5 K was three for both N_2_ and CH_4_, as dense polymer matrices are not subject to large changes in volume for moderate pressure increases.

### 3.3. Binary-Gas Uptakes in the Polyimides

In order to study the sorption of gases under conditions closer to industrial gas separations, the exposure of the polymer membranes to a mixed-gas reservoir was also examined [33]. In this case, the equilibrium condition between the gas phase mixture of fixed composition and the polymer + penetrants system is the same as before, but this now applies to each penetrant. For rigid gas molecules, this implies that the equality of the concentration and solubility ratios, i.e., *C*_pol_/*C*_gas_ = *S*_pol_/*S*_gas_ (Equation (7)), must hold for each type of penetrant.

The iterative technique for single-gas sorption, as described in Section 3.2., has previously been adapted to estimate a binary-gas uptake [56]. A detailed description has been given for the sorption of a binary CH_4_/N_2_ mixture in the 6FDA-mPDA, 6FDA-durene, and TR polyimides [59]. The main difference with the single-gas procedure is that, for a binary-gas system, the applied pressure is fixed and the numbers of each type of molecule inserted into the polymer matrix are iterated to convergence.

**Figure 4:**
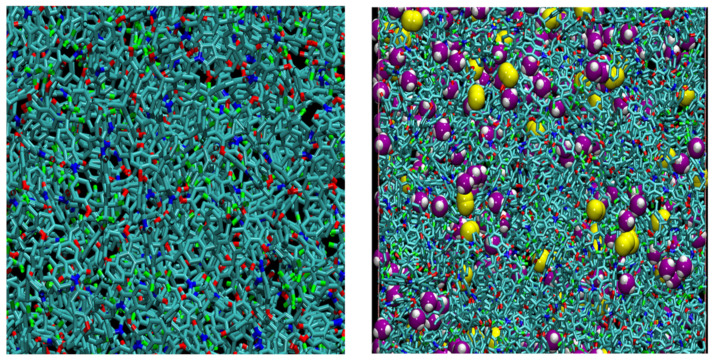
Close-up view of the pure 6FDA-BAPT at 338.5 K (**left**), and the 6FDA-BAPT mixed-gas CH_4_/N_2_ system at 338.5 K and *p* = 65.5 bar 6FDA-BAPT system at 338.5 K (**right**). For clarity, all hydrogens in the polymer are omitted. The polymer is presented using bonds with the following colour codes: C—cyan, F—lime green, O—red, and N—blue. The penetrant molecules are presented in VDW format. For methane: C—purple and H—white. For nitrogen: N—yellow. Image created using version 1.9.4 of *VMD* [87].

For the polymer membranes exposed to the binary mixture reservoir, the number of penetrants of both gas types to be initially inserted into the matrices was estimated from the single-gas uptake results. Each polyimide + N_2_ + CH_4_ system was relaxed under *NVT* conditions for 100 ps, and afterwards, *N**P**T* runs of at least 4 ns were conducted to allow the systems to reach their equilibrium size and density. The last 2 ns of these runs were used for subsequent analyses. To determine whether penetrants of each gas species had to be added or removed, a comparison of the solubility, *S*_gas_/*S*_pol_, and concentration, *C*_gas_/*C*_pol_, ratios for each gas was carried out. The absolute value of the difference between the two aforementioned ratios was iteratively minimised in order to reach convergence. In the systems under study, the difference between the concentration and solubility ratios was minimised within a maximum of five iterations in all models, taking into account errors in calculations. Snapshots of the 6FDA-BAPT homopolyimide both in the pure state as well as loaded with N_2_ and CH_4_ at 338.5 K and 65.5 bar are provided in Figure 4. The corresponding snapshots for 6FDA-mPDA/BAPT are provided in the Supplementary Materials.

### 3.4. Diffusion Coefficients in the Polyimides

As noted above, the permeation studies were carried out at 65.5 bar and 338.5 K for pure N_2_, pure CH_4_, and a binary 2:1 mixture of CH_4_/N_2_ in both matrices. To estimate their respective diffusion coefficients, the six simulations at 65.5 bar and 338.5 K were extended up to 15–20 ns with *N**P**T* MD, which allowed the Fickian regime for diffusion to be attained (see later). On these grounds, the self-diffusion coefficients, *D*, could be extracted using Einstein’s equation (Equation (8)):

(8) $$D=\underset{t\to \infty }{\lim}\frac{{\langle \left(r_{i}\left(t+t_{0}\right)-r_{i}\left(t_{0}\right)\right)}^{2}\rangle }{6t}$$

using the mean square displacements, *MSD* = <(***r***_i_ (*t* + *t*_0_) – ***r***_i_(*t*_0_))^2^>, of a given type of penetrant averaged over all time origins, *t*_0_, and over all penetrants of the same type.

## 4. Results and Discussion

### 4.1. Single-Gas Sorption of N_2_ and CH_4_ in the Triptycene-Based Polyimides

In this section, we present the results obtained from the pure-gas simulations at 338.5 K in which N_2_ and CH_4_ were gradually loaded separately in the polyimides’ matrices and the sorption isotherms that were obtained over the pressure range of 0–80 bar through the iterative method described in Section 3.2. In order to be consistent with experiments, the gas concentrations in the polymer at a gas reservoir pressure *p, C*_pol_(*p*), are given in terms of the volume that the number of gas molecules in the matrix, *n*_pol_(*p*), would occupy if they were an ideal gas at the standard temperature and pressure (STP) conditions, divided by the volume of the pure polymer volume, *V_0_.* These nominal concentrations, *C*_0_(*p*), do not take into account the limited volume swelling, which is very difficult to measure experimentally [88,89] (Equation (9)):

(9) $$C_{0}\left(p\right)=\frac{n_{\mathrm{pol}}\left(p\right)\;k_{B}\;T^{\mathrm{STP}}}{V_{0}\;p^{\mathrm{STP}}}$$

where *p*^STP^ = 1.01325 bar and *T*^STP^ = 273.15 K are the standard temperature and pressure, respectively. The nominal solubility coefficient is then defined as (Equation (10)):

(10) $$S_{c_{0}}\left(p\right)=\frac{C_{0}\left(p\right)}{p}$$

**Figure 5:**
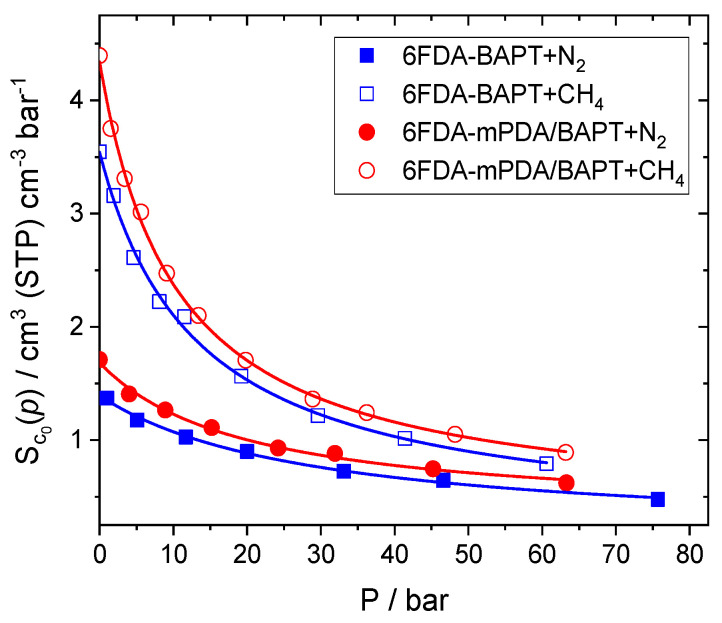
The nominal solubility coefficients for N_2_ (coloured symbols) and CH_4_ (white symbols) in 6FDA-BAPT (blue) and 6FDA-mPDA/BAPT (red) at 338.5 K as a function of the reservoir pressure, *p*. The smooth curves are fit to the dual-mode sorption model.

The nominal solubility coefficients are plotted with symbols for both gases and both matrices in Figure 5 as a function of *p*. The limiting values of the solubility coefficient for the penetrant molecule in the matrices can be obtained from (Equation (11)) [90]:

(11) $$\underset{p\to 0}{\lim}S_{c_{0}}\left(p\right)=\frac{S_{\mathrm{pol}}\;T^{\mathrm{STP}}}{T\;p^{\mathrm{STP}}}$$

In addition, the added smooth curves in Figure 5 are nonlinear least-squares regression fits to the dual-mode sorption (DMS) model (Equation (12)) [58]:

(12) $$S_{c_{0}}\left(p\right)=k_{D}+C_{H}^{\prime }\;\frac{b}{\left(1+bp\right)}$$

according to which gas sorption in glassy polymers is manifested both by a “hole-filling” mechanism described by the Langmuir sorption capacity, $C_{H}^{\prime }$, and the hole affinity, *b,* as well as by the dissolution into the dense regions obeying Henry’s law characterised by the constant *k*_D_. A drawback of the DMS model is that it is difficult to calculate its parameters from the known properties of the polymers and the dissolved gases [91]. However, the functional form of Equation (12) is often used to fit experimental single-gas uptake curves. Figure 5 clearly shows that, as in the experiment, the model results can be fitted by DMS too.

In accordance with other studies [56,59], methane is more soluble than nitrogen in both matrices. The block copolyimide exhibits consistently higher solubilities over the whole pressure range for both gases, which is consistent with the larger holes in 6FDA-mPDA/BAPT compared to 6FDA-BAPT (Figure 2 and Figure 3). Nevertheless, the differences between both matrices are rather small. This is expected since it is well known that polyimides usually have fairly close penetrant solubilities and that the separations of gases in these polymers are often mainly due to the diffusion selectivities [92].

The TPI analyses provide the weighted mean test particle insertion energy (Equation (13)):

(13) $$\langle \Delta \Phi \rangle =\frac{\left\langle \Delta \Phi \;\;Vexp\left(-\frac{\Delta \Phi }{k_{B}T}\right)\right\rangle }{\left\langle Vexp\left(-\frac{\Delta \Phi }{k_{B}T}\right)\right\rangle }$$

To ensure that the averages in Equation (13) represent the typical mean interaction energies that the probe molecule would have in the matrices, they can be weighted by the Boltzmann’s factor [59]. In general, the absolute values of the mean insertion energies for both systems follow the same hierarchy as the solubility coefficients, and indeed, that for nitrogen (≈15 kT is lower than that for methane (≈20 kT. They differ marginally between the two models.

From the TPI analyses, it is also possible to measure the fraction of the “significant accessible volume” (*SAV*) (also sometimes called “fraction of significant volume” (*FSV*) [89]), which is actually available for the insertion of a probe molecule. Unlike the geometric criterion used in Section 2.2, this approach is based on an energetic criterion, i.e., it estimates the available volume that is responsible for 99.9% of the solubility [60]. In the case of incompressible systems, the probability densities of the insertion energies, $\rho \left(\Delta \Phi \right),$ can be related to the solubility via (Equation (14)) [60]:

(14) $$\int_{-\infty }^{+\infty }\rho \left(\Delta \phi \right)exp\left(-\frac{\Delta \phi }{k_{B}T}\right)d\Delta \phi$$

The associated integrals of these functions reach a plateau relatively quickly as the Boltzmann’s weighting factor rapidly diminishes with the increase in insertion energy. The critical upper limit, Δ*Φ*_c_, for the solubility (by which the integrals are converged to more than 99.9%) is first determined. Integrating the probability density distribution functions up to this critical energy will then yield the fraction of *SAV* for the molecule being inserted (Equation (15)) [60]:

(15) $$SAV=\int_{-\infty }^{\Delta \Phi c}\rho \left(\Delta \Phi \right)d\Delta \Phi$$

**Figure 6:**
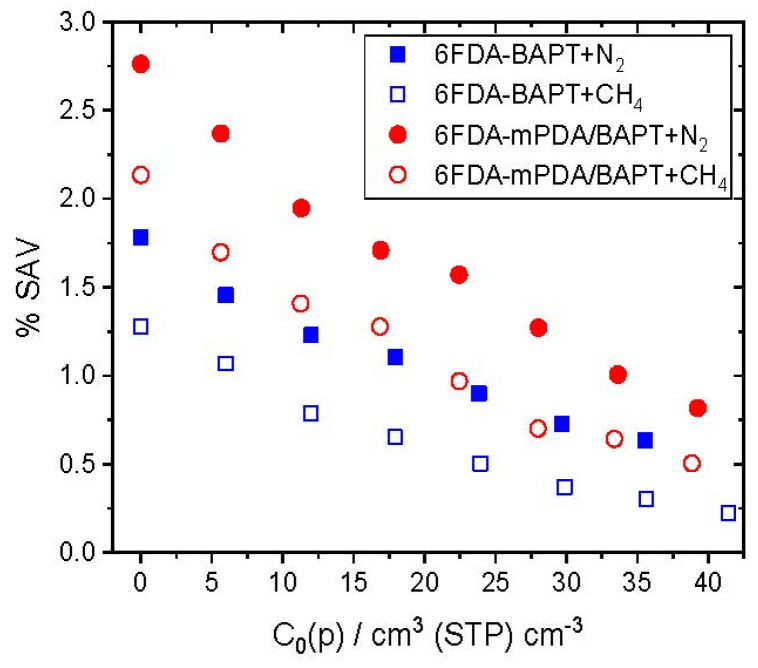
Percentage of significant accessible volume (%*SAV*) for the insertion of N_2_ (coloured symbols) and CH_4_ (white symbols) in 6FDA-BAPT (blue) and 6FDA-mPDA/BAPT (red) as a function of the penetrant nominal concentration, *C*_0_(*p*).

Given that this approach implicitly takes into account the interactions of each probe with its matrix, it allows for a better estimation of the volume really used by the probe molecule in question. Figure 6 presents the %*SAV* in both the pure matrices (*C*_0_(*p*) = 0) and in the polymer + penetrants systems as a function of the penetrant nominal concentrations over the whole range of *p* studied for the single-gas simulations.

The relative order of the %*SAV* between both polyimides is in accordance with the predictions from the phantom sphere approach for large probe radii in pure polymers (Figure 2 and Figure 3). This shows, again, that the incorporation of the mPDA diamine into the block copolyimide results in a significant gain in accessible volume with respect to 6FDA-BAPT. The *%SAV* is higher for the less soluble N_2_ penetrant, which means that it needs to reach more sites in the matrix than CH_4_ to account for its solubility. On the other hand, the latter favours those sites with the most favourable energetic interactions. The *%SAV* drops with penetrant concentrations as sites are progressively filled up.

### 4.2. Volume Dilations upon Single-Gas Sorption

Gas-sorption-induced volume dilation effects are difficult to capture experimentally [67,68]. In contrast, volume swelling can be extracted easily in MD simulations in 3D periodic boundary conditions by calculating the volume change in the simulation box with respect to the pure polymer volume, *V*_0_ [56,93].

To our knowledge, there are no available volume dilation data for N_2_ or CH_4_ in triptycene-based polyimides. However, a few studies examined volume swelling in glassy polymers due to these penetrants [88,94,95]. They report CH_4_-induced volume swelling up to 2% at pressures from 60 to 70 bar [88,94], whereas the volume changes induced by N_2_ in a glassy copolymer reach 0.6% at 30 bar [95]. Methane can, thus, be considered as moderately plasticizing, whereas nitrogen is non-plasticizing.

In a previous simulation of the N_2_/CH_4_ gas pair in fluorinated polyimides [56], CH_4_-induced volume dilation reached ~5% at pressures approaching 70 bar, whereas there was limited swelling upon N_2_ sorption. The dilation effects in a 6FDA-6FpDA polyimide were found to be ~2.6% for CH_4_ and ~1.4% for N_2_ at 60 bar [96]. Another work addressing the sorption of N_2_ and CH_4_ in thermally rearranged (TR) polymers reported a volume swelling for the 6FDA-bisAPAF polyimide precursor of ~3.6% for CH_4_ and ~1.8% for N_2_ at pressures of ~63 and ~73 bar, respectively, whereas both gases induced negligible dilations for the TR structure [59]. In the present case, N_2_ and CH_4_ induce volume changes in 6FDA-BAPT of about 1.3% and 1.8%, respectively, at the highest gas loadings, i.e., at feed pressures approaching 60–70 bar. These findings support experimental work demonstrating the high plasticisation resistance for triptycene-based polyimides [39,40] and indicate that 6FDA-BAPT is less prone to volume dilation than other non-thermally rearranged 6FDA-based polyimides [55]. For the 6FDA-mPDA/BAPT copolyimide, N_2_ sorption induces negligible swelling, i.e., less than 0.4%, which is probably due to its larger holes (Figure 2) but CH_4_ swells the matrix up to 3% at a pressure of 64 bar. The latter can be attributed to the mPDA diamine structure, which is known to be less resistant to plasticisation than the more rigid and bulky BAPT counterpart [62].

### 4.3. Binary-Gas Sorption of a 2:1 CH_4_/N_2_ Mixture in the Triptycene-Based Polyimides

When in equilibrium with a 2:1 binary-gas CH_4_/N_2_ mixture at 65.5 bar and at 338.5 K, the mixed-gas compositions in the polyimide models were obtained via the implementation of the method described in Section 3.3. The nominal concentrations, *C*_0_, issued from the MD simulations are presented in Table 1, along with the *C*_0_ predictions obtained from the extension of the DMS model to binary mixtures [97,98] (Equation (16)):

(16) $$C_{0_{A}}\left(p\right)=K_{D_{A}}p_{A}+C_{H_{A}}^{\prime }\left(\frac{b_{A}p_{A\;\;}}{1+b_{A}p_{A}+b_{B}p_{B\;\;}}\right)$$

where A and B are the two different gases, and *p*_A_ and *p*_B_ are the partial pressures of each gas. The constants for each gas are estimated from the DMS fits to the single-gas sorption curves (Figure 5).

Slight differences in mixed-gas compositions are observed between 6FDA-BAPT and 6FDA-mPDA/BAPT. Both gases exhibit higher concentrations in the block copolyimide, in accordance with the single-gas uptake curves presented in Figure 5. The molar gas ratios in the polymer phases are higher than 4:1, deviating both from the ratios of CH_4_ and N_2_ molecules based on the pure-gas solubilities (Figure 5), as well as from the 2:1 molar ratio of the pure binary-gas phase. A similar trend is observed for the nominal *C*_0_ concentrations obtained from the DMS theory extended to binary mixtures (Equation (16)), which are in reasonable agreement with the simulated *C*_0_ (Table 1). The higher sorption of methane over nitrogen, which is expected from the higher solubility of the former and from its higher molar fraction in the binary-gas phase, leads to a similar volume swelling of ~2% for both matrices, with the degree of volume swelling being intermediate between the single-gas swellings (Section 4.2).

These results lie in accordance with analogous studies on N_2_/CH_4_ mixed-gas sorption in thermally rearranged polymer membranes [59]. The data in Table 1 also demonstrate the robustness of the DMS theory for binary mixtures as it allows for a fairly satisfactory prediction of the penetrant concentrations in the polymer phase without the need to conduct mixed-gas simulations. However, the adequacy of the simulated *C*_0_ is both matrix- and penetrant-dependent, and in the case of the block copolyimide, the DMS theory does underestimate the ratio of the equilibrium gas concentrations by ~10–20%.

To examine possible preferential interactions between the polymer and one of both gases, intermolecular radial distribution functions, g_inter_(r), were calculated between the nitrogen centre of mass, the methane carbon and the different types of polymer atoms, with averages being taken over all frames of the production runs. These functions were, thus, analysed in the case of all the mixed-gas systems; examples are given for the 6FDA-mPDA/BAPT + N_2_ + CH_4_ system comprising the gas concentrations listed in Table 1.

**Figure 7:**
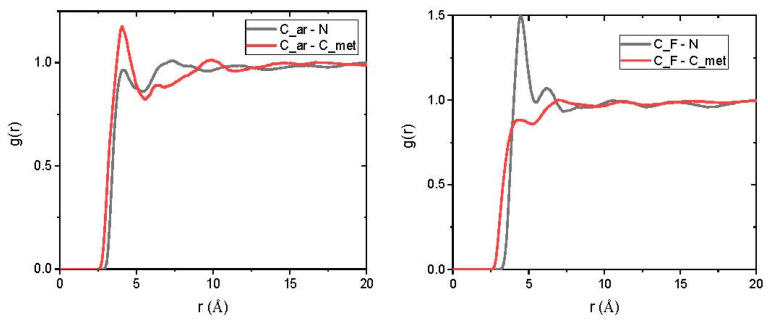
Intermolecular radial pair distribution functions between the centre of mass of a nitrogen molecule, N, or the methane carbon, C_met, and either (**left**) the dianhydride aromatic carbon or the CF_3_ group carbon (**right**).

As can be inferred from Figure 7, methane shows a higher probability of interaction with the polymer aromatic carbon than nitrogen, whereas the inverse tendency is observed when the gases interact with the fluorocarbon group. This implies that not all polymer sites interact with the gas components in the same manner.

### 4.4. Single-Gas vs. 2:1 Binary-Gas CH_4_/N_2_ Gas Diffusivities, Permeabilities, and Permselectivities in the Triptycene-Based Polyimides

This section compares the diffusion, permeation, and separation abilities for both mixed-gas systems at 65.5 bar and 338.5 K with the pure-gas systems. As the simulations for the latter were performed at a fixed number of penetrants, the pure-gas systems closest in pressure to 65.5 bar were used for the comparison.

**Figure 8:**
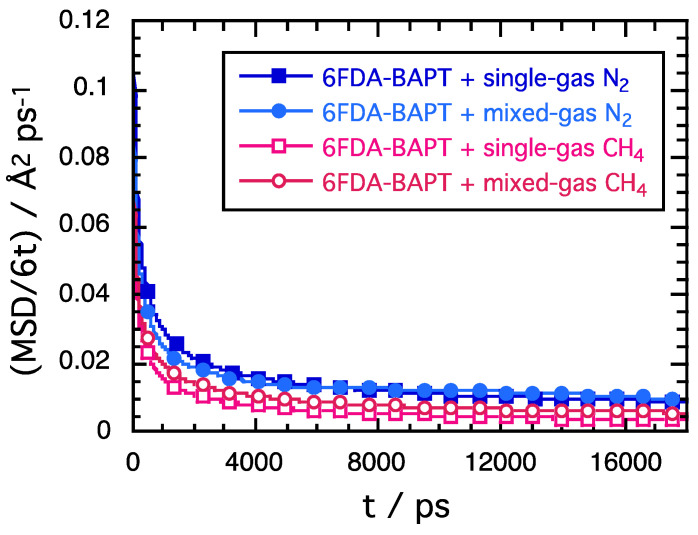
The MSD/6t curves as a function of simulation time, *t*, for single-gas N_2_, single-gas CH_4_, and mixed-gas N_2_ and CH_4_ at 65.5 bar and 338.5 K. The plateau values allow for the estimation of the diffusion coefficients.

As explained in Section 3.4, the gas diffusion coefficients were extracted from the MSD of the gas centres of mass, which were calculated from MD trajectories extrapolated up to at least 15 ns. Figure 8 presents the MSD/6*t* vs. simulation time, *t*, for the gases in the 6FDA-BAPT matrix and shows that each MSD/6*t* reaches a plateau value, which can provide a reasonable estimation of *D* (Equation (8)). This is also the case for 6FDA-mPDA/BAPT, although the plateau values were attained a bit faster, as the penetrants were more mobile in the copolyimide.

The gas permeabilities, *P*, were estimated using Equation (1) from the product of the *S*_c_ solubility coefficients extracted from Equation (2) and the diffusion coefficients, *D*, obtained from Equation (8). The separation factors or permselectivities, *α*, were obtained from Equation (3). Table 2 lists the gas solubility, diffusion, and permeability in the mixed-gas systems at 65.5 bar together with the corresponding data of the single-gas systems at the feed pressures closest to this value. The mixed N_2_/CH_4_ separation factors are obtained from the binary-gas runs, and the pure N_2_/CH_4_ separation factors are the ideal permselectivities from the single-gas runs. We note here that the *S*_c_ are reported in units of cm^3^(STP)/cm^3^(cm Hg) as this is a straightforward way to calculate the *P* in Barrer afterwards (1 Barrer = 10^−10^ cm^3^(STP) cm^2^/(cm^3^ s (cm Hg)). *D* are provided in units of cm^2^ s^−1^. The maximum errors on *S*_c_ are 0.0001 cm^3^(STP)/cm^3^(cm Hg), while those on *D* are larger due to the plateau values in Figure 8. They are all typically of the order of 10^−7^ cm^2^ s^−1^. As such, only the specific errors on *P* and on the separation factors are reported in Table 2.

Upon mixing, there is a slight decrease in the solubility coefficients, *S*_c_, for N_2_ in both matrices, which is to be expected because of the competition with the more soluble CH_4_. Indeed, the *S*_c_ of CH_4_ increases simultaneously from ~1.5 in the single gas to ~2.5 larger than for N_2_ in the mixed-gas systems. The CH_4_/N_2_ solubility selectivity is, thus, enhanced under mixed-gas conditions. However, as observed in the sorption isotherms (Figure 5), the *S*_c_ for both gas components exhibits only small differences between both 6FDA-BAPT and 6FDA-BAPT/mPDA. Since the permeabilities, *P*, are quite different, this suggests that the kinetic aspect also plays a role [37], which is similar to analogous studies on fluorinated polyimides [56].

N_2_ diffuses faster than CH_4_, as expected from its slightly smaller kinetic diameter [27]. In absolute terms, the fastest diffusivities, *D*, and, hence, the highest gas permeabilities, *P*, are observed in the 6FDA-BAPT/mPDA copolyimide. Indeed, the incorporation of the mPDA diamine induces an increase in the significant accessible volume for both gas penetrants, thus enhancing their overall mobilities by a factor of 2–3 with respect to 6FDA-BAPT. However, in both matrices, the N_2_/CH_4_ diffusion selectivities are all less than ~2.

The ideal permselectivity of 6FDA-BAPT for the N_2_/CH_4_ pair has been reported experimentally to be ~1.8 at *T* = 35° and *p* = 9 bar. Although the conditions are different, this is quite coherent with the ideal permselectivity of our model [40] (Table 2). Due to its higher available volume, 6FDA-mPDA/BAPT seems to lose its permselectivity under single-gas conditions.

However, both matrices behave in different ways in the mixed-gas systems. The enhancements in solubility selectivities lead to larger CH_4_ and lower N_2_ permeabilities. This behaviour has also been detected in earlier experimental studies [97,99], as well as in simulations of fluorinated and TR polyimides [56,59]. Both triptycene-based polyimides, thus, favour the transport of methane over that of nitrogen under the mixed-gas conditions studied (2:1 CH_4_:N_2_ binary mixtures at 65.5 bar and 338.5 K). It is, thus, important to test gas mixtures in addition to single-gas studies [33].

## 5. Conclusions

MD simulations were performed to compare the transport of N_2_ and CH_4_ in two triptycene-based polyimides. The first one was the 6FDA-BAPT homopolyimide, and the second one was a block 6FDA-mPDA/BAPT copolyimide, combining the high FFV of the triptycene diamine with the mPDA diamine, with the latter expected to be more selective. The densities of the pure polyimide models were found to be in agreement with experiment. However, the void-space analyses revealed that, while the free volume consists of small-to-medium holes in 6FDA-BAPT, there are more medium-to-large holes in 6FDA-mPDA/BAPT. Indeed, the 6FDA-mPDA block with a molecular weight of 15,000 g mol^−1^ is probably not long enough to induce a selective behaviour, and it further disrupts the 6FDA-BAPT block, hence leading to a copolyimide with more free volume.

Following the generation of the polymer matrices, a single-gas sorption technique iterating the pressure for a constant number of penetrants was applied to obtain the sorption isotherms for N_2_ and CH_4_ over a 0–70 bar range at 338.5 K. Both gases were found to be more soluble in the block copolyimide with a higher affinity for methane. This was confirmed by the fraction of significant accessible volume (*%SAV*), which showed that CH_4_ favours sites with the most favourable energetic interactions, unlike the lower-solubility N_2_, which probes more sites in the matrices. 6FDA-BAPT swelled less than 2% at the highest gas loadings, while 6FDA-mPDA/BAPT swelled up to 3% due to its less resistant mPDA diamine [39].

An adapted mixed-gas sorption technique iterating the number of gas penetrants for a constant external gas pressure was used to estimate the mixed-gas equilibrium concentrations of a 2:1 CH_4_/N_2_ mixture at 338.5 K and 65.5 bar in both matrices. The molar CH_4_/N_2_ ratios in the polymer phases were found to be higher than 4:1, i.e., with an enhancement in the solubility selectivity in favour of CH_4_. N_2_ and CH_4_ diffusivities were probed via the mean square displacements. Although diffusion was faster for N_2_ and generally higher in 6FDA-BAPT/mPDA, the diffusion selectivities were not significantly different from each other. As such, the higher solubility selectivities meant that the triptycene-based polyimides both favoured, to a similar extent, the transport of methane over that of nitrogen under the conditions studied.

Concerning the permselectivity performance of the triptycene-based PIs examined in this study as compared to other 6FDA-based PI membranes previously examined by our group, it appears that all polymer matrices behave in different ways in the mixed-gas systems compared to the single-gas case. 6FDA-mPDA/BAPT is highly permeable to methane in the mixed-gas case, which is similar to the behaviour observed in a thermally rearranged TR-PBO polyimide [59]. Since the mPDA diamine is methane-selective, when it is combined with nitrogen-selective diamines, such as the durene diamine, it induces a reduction in the nitrogen-selectivity of the latter [56]. Finally, the BAPT polyimide appears reasonably more resistant to plasticisation compared to the mPDA and durene diamines, with TR-PBO being the only polymer matrix that undergoes lower volume dilation under the same operating conditions.

## Figures and Tables

**Table 1:** Optimal numbers of each type of penetrant in the polyimide matrices when in equilibrium with 2:1 binary-gas CH_4_/N_2_ mixture at 65.5 bar and 338.5 K. Theoretical estimates of the nominal concentrations in units of cm^3^(STP) cm^−3^ are also shown together with the average volume dilations.

Polyimide	Gas	No. of Gas Molecules	*C*_0_ (Simulation)	*C*_0_ (Equation (16))	%Volume Swelling
**6FDA-BAPT**	**CH_4_**	331	39.756 ± 0.010	37.69	1.99 ± 0.04
	**N_2_**	71	8.527 ± 0.002	8.54	
**6FDA-mPDA/BAPT**	**CH_4_**	400	45.926 ± 0.002	42.29	2.10 ± 0.07
	**N_2_**	86	9.680 ± 0.001	11.81	

**Table 2:** Average permeabilities, *P*; solubility coefficients, *S*_c_; diffusion coefficients, *D*; and selectivities, α, for N_2_ and CH_4_ in the single- and mixed-gas simulations conducted at 338.5 K. The average feed pressure of the single-gas systems is also given. For the mixed-gas systems, the pressure is 65.5 bar. *P* are expressed in Barrer, *S*_c_ in cm^3^(STP)/cm^3^(cm Hg), and *D* in cm^2^ s^−1^.

Polyimide			6FDA-BAPT	6FDA-mPDA/BAPT
	*p*/bar		66.0	63.3
**N_2_**	Single gas	*P*	59 ± 7	122 ± 10
		$S_{c}$	0.0062	0.0081
		*D*	9 × 10^−7^	15 × 10^−7^
	Mixed gas	*P*	50 ± 6	107 ± 7
		$S_{c}$	0.0050	0.0057
		*D*	10 × 10^−7^	19 × 10^−7^
	*p*/bar		65.0	65.0
**CH_4_**	Single gas	*P*	44 ± 11	140 ± 14
		$S_{c}$	0.0108	0.0120
		*D*	4 × 10^−7^	12 × 10^−7^
	Mixed gas	*P*	74 ± 13	146 ± 15
		$S_{c}$	0.0126	0.0134
		*D*	6 × 10^−7^	11 × 10^−7^
***α*** **(N_2_/CH_4_)**	Single gas		1.3 ± 0.5	0.9 ± 0.2
	Mixed gas		0.7 ± 0.2	0.7 ± 0.1

## Data Availability

The data presented in this study are available on request from the corresponding author. The data are not publicly available due to restrictions imposed by the contract number 6600025762.

## Supplementary Materials

The following supporting information can be downloaded at: https://www.mdpi.com/article/10.3390/polym15183811/s1.
