# Supplementary material for: A Molecular Dynamics Study of Single-Gas and Mixed-Gas N2 and CH4 Transport in Triptycene-Based Polyimide Membranes
Source: Polymers (Basel). 2023 Sep 18;15(18):3811. doi: 10.3390/polym15183811 (PMC10535442; doi:10.3390/polym15183811)
Supplement: Supplementary file 1 [file polymers-15-03811-s001.zip › polymers-2555193-supplementary.pdf]

polymers

MDPI

Supporting Information

A Molecular Dynamics Study of Single-Gas and Mixed-Gas N_2 and CH_4 Transport in Triptycene-Based Polyimide Membranes

Ioannis Tanis [1,*,†], David Brown [1], Sylvie Neyertz [1], Milind Vaidya [2], Jean-Pierre Ballaguet [2], Sebastien Duval [2] and Ahmad Bahamdan [2]

[1] Univ. Savoie Mont Blanc, Univ. Grenoble Alpes, CNRS, Grenoble INP, LEPMI, 38000 Grenoble, France; david.brown@univ-smb.fr (D.B.); sylvie.neyertz@univ-smb.fr (S.N.)
[2] Saudi Aramco, Research & Development Center, P.O. Box 62, Dhahran 31311, Saudi Arabia; milind.vaidya@aramco.com (M.V.); peterball128@yahoo.fr (J.-P.B.); sebastien.duval@aramco.com (S.D.); ahmad.bahamdan@aramco.com (A.B.)
* Correspondence: ioannis.tanis77@gmail.com
† Current address: Siemens Digital Industries Software, 2595 BN The Hague, The Netherlands.

S1. Methane and nitrogen force field parameters

The symmetrical two-center Lennard-Jones plus point quadrupole pair potential (2CLJQ) from Vrabec et al. [1] was employed for the description of N_2. It is a rigid model which considers two identical Lennard-Jones (LJ) sites ($s_{N-N} = 3.3211$ Å, $e_{N-N} / k_B = 34.897$) a distance 1.0464 Å apart. An associated quadrupole moment is modeled by placing partial charges $q_N = -0.5474$ on the LJ sites and a balancing counter charge at the centre of mass of the molecule, $-2q_N$. The dynamics of such systems with an interaction site not located at a mass point were solved within the framework of special constraints, as explained by Ciccotti *et al.*[2]. This potential was found to reproduce a number of thermophysical properties of nitrogen at the boiling point such as the gas and liquid density and the heat of vaporization [3].

Methane was also represented by a fully atomistic model. All potential parameters, including partial charges, were obtained from the flexible methane model of Yin and co-workers [4] ($l_{C-H} = 1.111$Å , $s_{C-C} = 3.7595$Å , $e_{C-C} / k_B = 47.80$, $s_{H-H} = 2.3876$Å, $e_{H-H} / k_B = 8.5546$; $q_C = -0.36$, $q_H = 0.09$). However, to ensure a rigid tetrahedral representation, we considered CH_4 as an H_b-CH_{a2}-H_b molecule where the midpoint of the H_a-H_a vector remains on the bisector of the H_b-C-H_b bending angle and the aforementioned vector is maintained perpendicular to the vector joining the H_b hydrogen atoms. The latter were considered as 'bonded' at a distance $l_{H_b-H_b} = 1.8142$Å. All bond lengths were constrained using the SHAKE algorithm [2] and special CH_2 constraints were used to render the methane molecules completely rigid, as explained above. This allowed the integration of the equations of motion using a time step of 1 fs with no problems of equipartition of kinetic energy between the different modes. The assessment of the rigid model showed satisfactory agreement with experimental data regarding the thermophysical properties mentioned earlier [3].

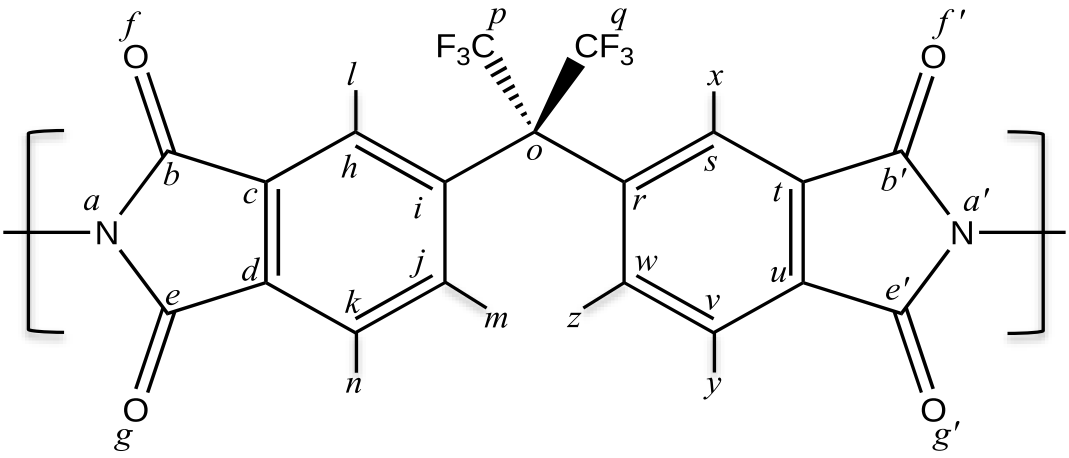

Figure S1. Structure of the 6FDA fragment. Letters in italics distinguish the different atoms whose charges are given in Table S1.

Table S1. Partial charges, q/e, on the atoms in the 6FDA fragment. An asterisk implies the same charge as the symmetrically equivalent atom.

	a	***b***	***c***	***d***	***e***	***f***	***g***	***h***	***i***	***j***	***k***
BAPT-**6FDA**-BAPT	-0.3812	0.5697	-0.0677	-0.1345	0.5965	-0.4461	-0.4568	-0.1467	0.2291	-0.1428	-0.0800
mPDA-**6FDA**-mPDA	-0.4475	0.6045	-0.0866	-0.0963	0.5985	-0.4502	-0.4542	-0.1499	0.2291	-0.1488	-0.0964
BAPT-**6FDA**-mPDA	-0.3473	0.5792	-0.1579	-0.0587	0.5908	-0.4807	-0.4881	-0.0833	0.1614	-0.0839	-0.1486
mPDA-**6FDA**-BAPT	-0.3467	0.5798	-0.1573	-0.0581	0.5914	-0.4801	-0.4875	-0.0827	0.1620	-0.0833	-0.1480
	l	*m*	*n*	*o*	*p*	*q*	*r*	*s*	*t*	*u*	*v*
BAPT-**6FDA**-BAPT	0.1340	0.1378	0.1344	-0.4578	0.3949	-0.1093	*	*	*	*	*
mPDA-**6FDA**-mPDA	0.1331	0.1420	0.1395	-0.4088	0.3855	-0.1085	*	*	*	*	*
BAPT-**6FDA**-mPDA	0.1416	0.1287	0.1436	-0.4992	0.4524	-0.1220	0.1988	-0.1185	-0.0845	-0.1336	-0.0922
mPDA-**6FDA**-BAPT	0.1422	0.1293	0.1442	-0.4980	0.4530	-0.1214	0.1994	-0.1179	-0.0839	-0.1330	-0.0916
	w	*x*	*y*	*z*	*a′*	*b′*	*e′*	*f ′*	*g′*		
BAPT-**6FDA**-BAPT	*	*	*	*	*	*	*	*	*		
mPDA-**6FDA**-mPDA	*	*	*	*	*	*	*	*	*		
BAPT-**6FDA**-mPDA	-0.1357	0.1466	0.1361	0.1407	-0.2275	-0.4655	0.5759	-0.4655	-0.4781		
mPDA-**6FDA**-BAPT	-0.1351	0.1472	0.1367	0.1413	-0.2269	0.5125	0.5765	-0.4649	-0.4775		

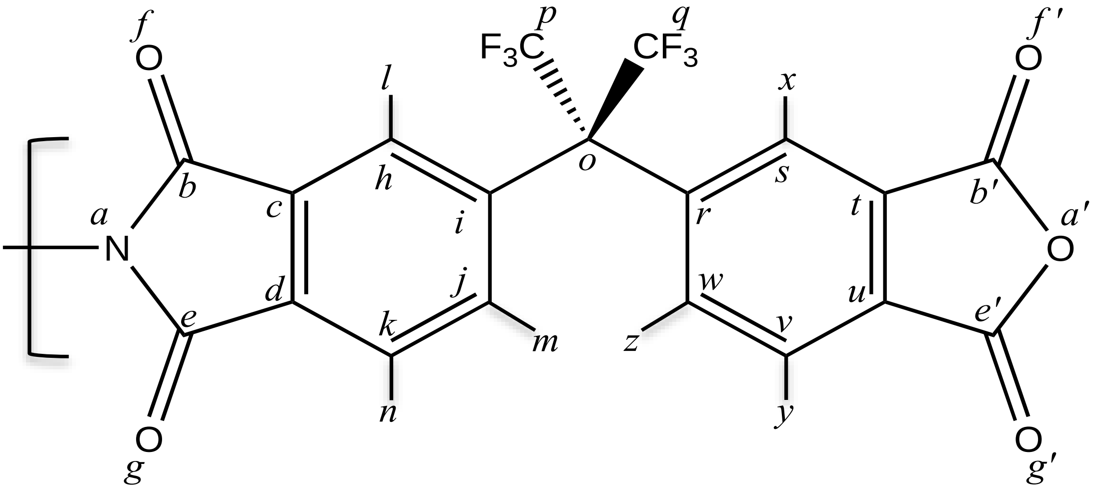

Figure S2. Structure of the 6FDA end fragment. Letters in italics distinguish the different atoms whose charges are given in Table S2.

Table S2. Partial charges, q/e, on the atoms in the 6FDA end fragment.

	a	***b***	***c***	***d***	***e***	***f***	***g***	***h***	***i***	***j***	***k***
BAPT-**6FDA_end**	-0.2979	0.6346	-0.2172	-0.0475	0.5318	-0.4825	-0.4672	-0.0909	0.3792	-0.2117	-0.1309
mPDA-**6FDA_end**	-0.4054	0.5418	-0.0342	-0.1307	0.5915	-0.4249	-0.4487	-0.1945	0.2658	-0.1543	-0.0877
	l	*m*	*n*	*o*	*p*	*q*	*r*	*s*	*t*	*u*	*v*
BAPT-**6FDA_end**	0.1352	0.1385	0.1476	-0.5927	0.4292	-0.1139	0.2913	-0.1203	-0.1375	-0.1377	-0.1135
mPDA-**6FDA_end**	0.1380	0.1448	0.1388	-0.5362	0.4526	-0.1212	0.3258	-0.1749	-0.0839	-0.1566	-0.0985
	w	*x*	*y*	*z*	*a′*	*b′*	*e′*	*f ′*	*g′*		
BAPT-**6FDA_end**	-0.2145	0.1281	0.1472	0.1428	-0.3516	0.6704	0.6854	-0.4490	-0.4572		
mPDA-**6FDA_end**	-0.1335	0.1293	0.1450	0.1308	-0.4282	0.6771	0.7000	-0.4239	-0.4317		

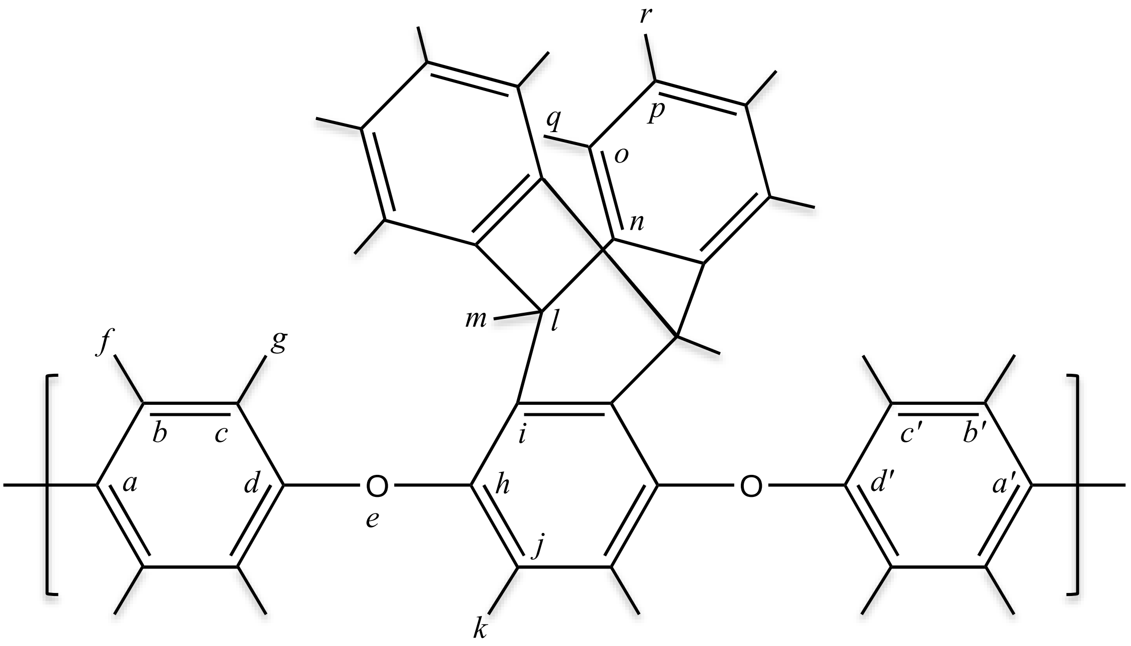

Figure S3. Structure of the BAPT fragment. Letters in italics distinguish the different atoms whose charges are given in Table S3.

Table S3. Partial charges, q/e, on the atoms in the BAPT fragment. An asterisk implies the same charge as the symmetrically equivalent atom.

	a	***b***	***c***	***d***	***e***	***f***	***g***	***h***	***i***
	0.2483	-0.2206	-0.2053	0.3332	-0.3522	0.1770	0.1498	0.2614	-0.0999
	j	*k*	*l*	*m*	*n*	*o*	*p*	*q*	*r*
6FDA-**BAPT**-6FDA	-0.2471	0.1686	0.1998	0.0273	-0.0035	-0.1707	-0.1222	0.1136	0.1203
	a′	*b′*	*c′*	*d′*					
	*	*	*	*					

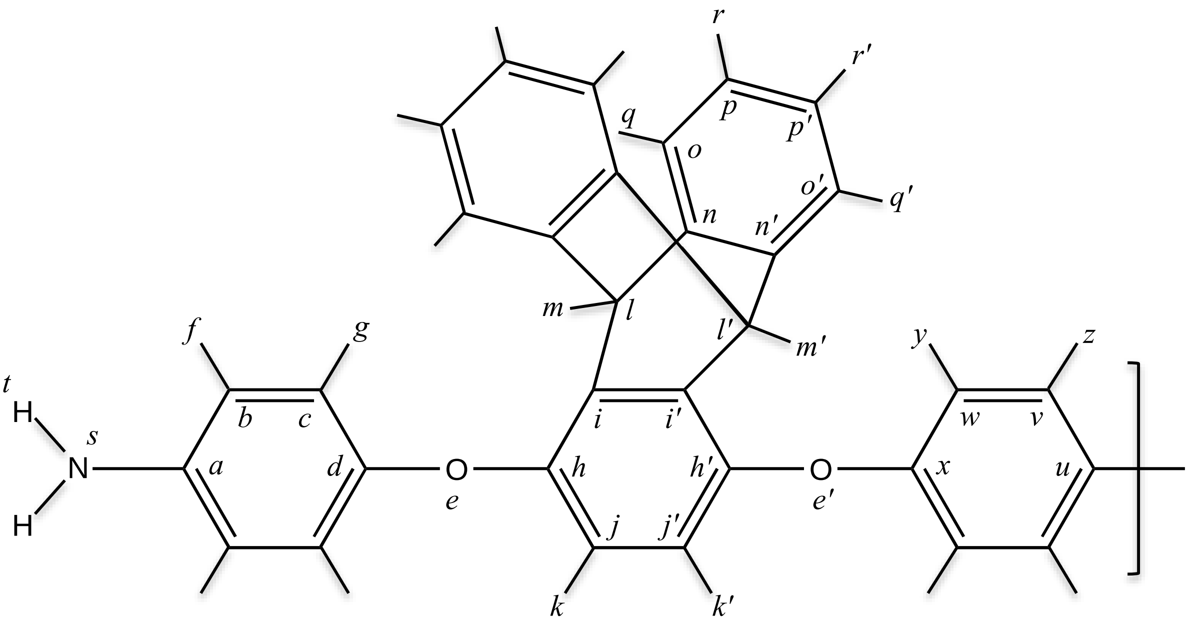

Figure S4. Structure of the BAPT start fragment. Letters in italics distinguish the different atoms whose charges are given in Table S4.

Table S4. Partial charges, q/e, on the atoms in the BAPT start fragment.

	a	***b***	***c***	***d***	***e***	***f***	***g***	***h***	***i***
	0.3455	-0.2613	-0.2193	0.3237	-0.3195	0.1593	0.1562	0.1809	-0.0371
	j	*k*	*l*	*m*	*n*	*o*	*p*	*q*	*r*
	-0.1930	0.1324	0.0751	0.0561	0.0141	-0.1731	-0.1177	0.1218	0.1170
	s	*t*	*u*	*v*	*w*	*x*	*y*	*z*	*e'*
**BAPT_start**-6FDA-BAPT	-0.7809	0.3374	0.2982	-0.2517	-0.1718	0.2687	0.1389	0.1754	-0.3410
	h'	*i'*	*j'*	*k'*	*l'*	*m'*	*n'*	o'	*p'*
	0.2896	-0.0298	-0.2873	0.1828	-0.2206	0.1505	0.0809	-0.1296	-0.1454
	q'	r'							
	0.1109	0.1222							

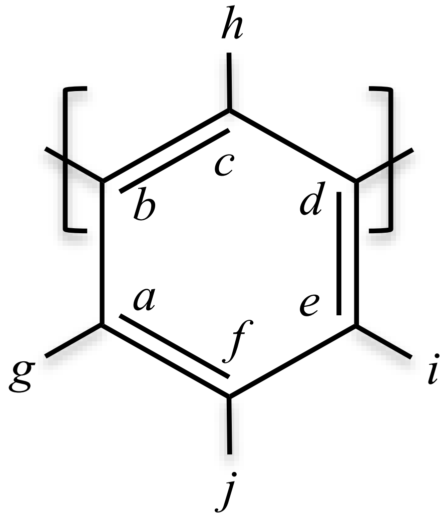

Figure S5. Structure of the mPDA fragment. Letters in italics distinguish the different atoms whose charges are given in Table S5.

Table S5. Partial charges, q/e, on the atoms in the mPDA fragment. An asterisk implies the same charge as the symmetrically equivalent atom.

	a	*b*	*c*	*d*	*e*	*f*	*g*	*h*	*i*	*j*
6FDA-**mPDA-**6FDA	-0.3793	0.4667	-0.4975	*	*	0.0508	0.1893	0.2387	*	0.1098

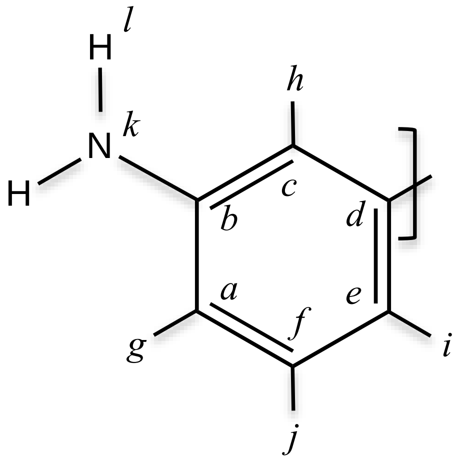

Figure S6. Structure of the mPDA start fragment. Letters in italics distinguish the different atoms whose charges are given in Table S6.

Table S6. Partial charges, q/e, on the atoms in the mPDA end fragment.

	a	*b*	*c*	*d*	*e*	*f*	*g*	*h*	*i*	*j*
**mPDA_start-**6FDA-mPDA	-0.3289	0.4834	-0.4748	0.4020	-0.3559	-0.0176	0.1632	0.2099	0.1764	0.1230
	k	*l*								
	-0.7896	0.3250								

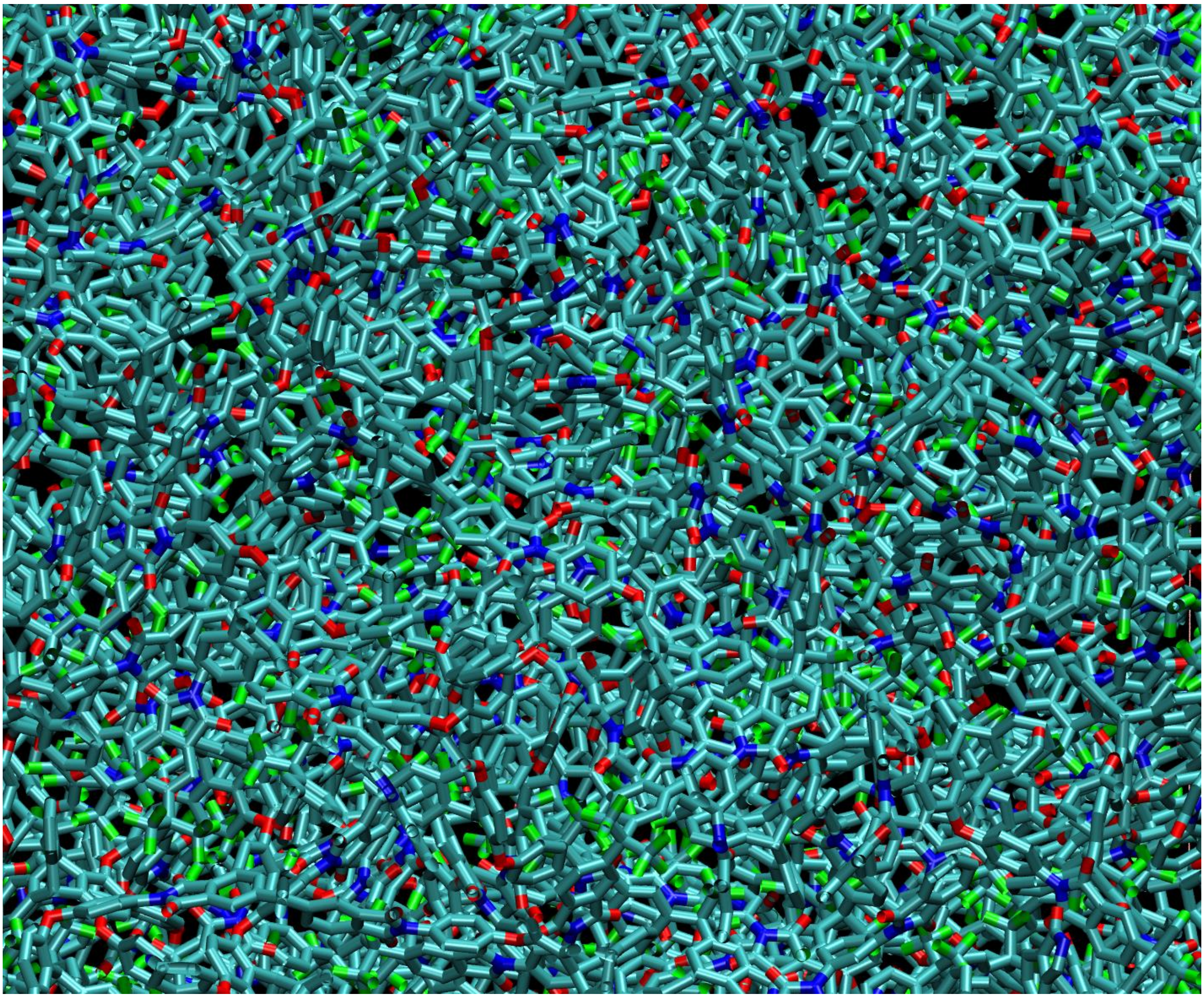

Figure S7. Close-up view of the pure (6FDA-mPDA)/(6FDA-BAPT) copolymer system at 338.5 K. For clarity all hydrogens have been omitted. The polymer is presented using bonds with the colour code following: C = cyan, F = lime green, O = red and N = blue. Image created using version 1.9.4 of *VMD* [1].

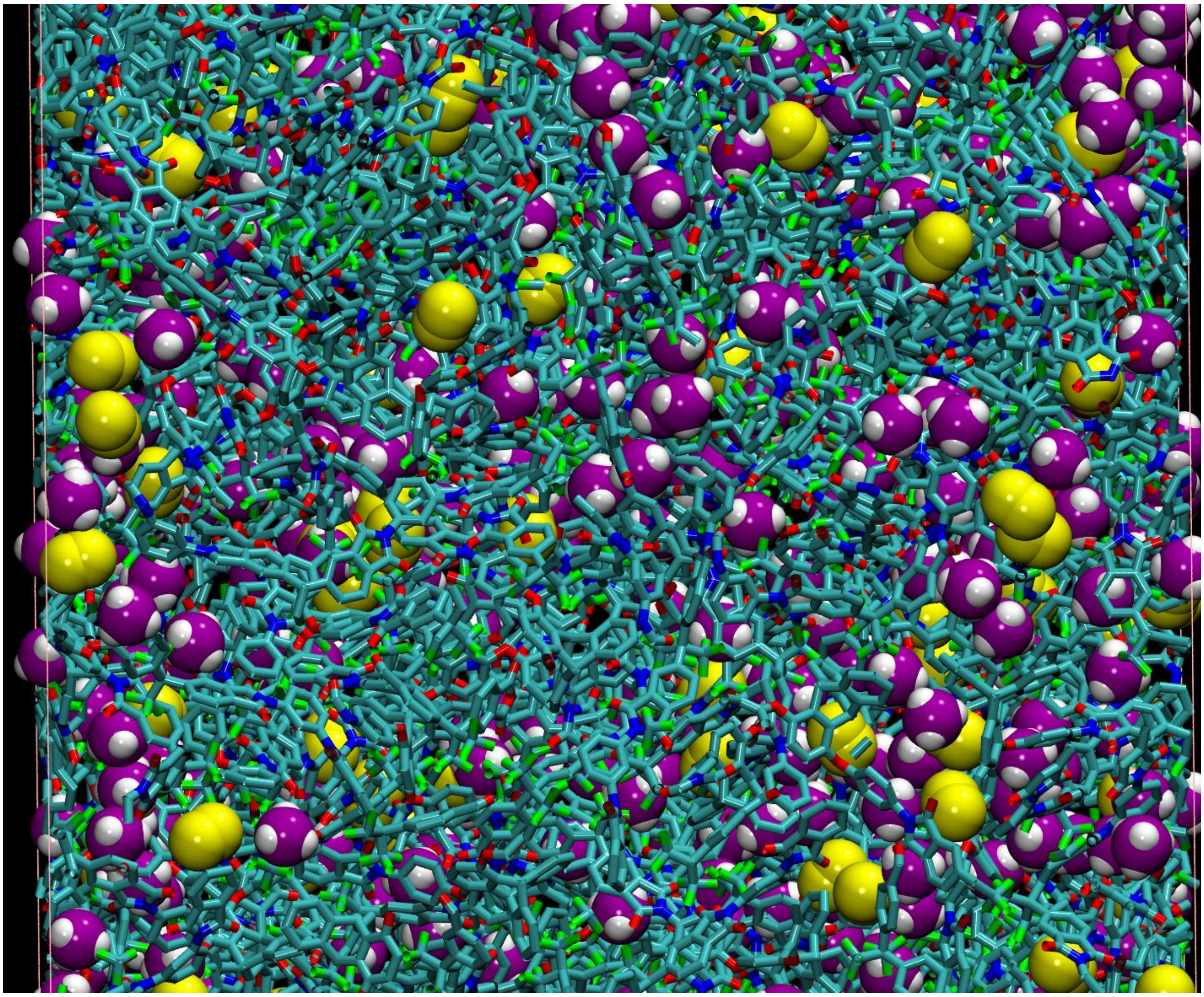

Figure S8. Close-up view of the (6FDA-mPDA)/(6FDA-BAPT) mixed gas CH_4/N_2 system at 338.5 K and p = 65.5 bar. For clarity all hydrogens in the polymer have been omitted. The polymer is presented using bonds with the colour code following: C = cyan, F = lime green, O = red and N = blue. The penetrant molecules are presented in VDW format. For methane: C = purple and H = white. For nitrogen: N = yellow. Image created using version 1.9.4 of *VMD* [1].

Table S7. Correspondence between the atom-type numbers, the names used in *gmq* and the symbols used in the TRIPOS 5.2 forcefield.

Atom Type Number	Atom Name in *gmq*	Atom Type in TRIPOS
1	C_1	C_3
2	C_{F1}	C_3
3	C_{ar1}	C_{ar}
4	C_{ket}	C_2
5	O_{ket}	O_2
6	N	N_{am}
7	F_1	F
8	C_{ar2}	C_{ar}
9	C_2	C_3
10	C_{F2}	C_3
11	F_2	F

12	C_{CH3}	C_3
13	H_{all}	H
14	H_{CH3}	H
15	H_{term}	H
16	O_3	O_3
17	N_3	N_3
18	O_{tr}	O_3
19	C_{artr}	C_{ar}
20	O_{term}	O_3
21	C_{arfda}	C_{ar}
22	C_{fda}	C_3
23	C_{arbapt}	C_{ar}
24	O_{bapt}	O_3
25	C_{bapt}	C_3

References

1. Vrabec, J.; Stoll, J.; Hasse, H. A Set of Molecular Models for Symmetric Quadrupolar Fluids. *The Journal of Physical Chemistry B* **2001**, *105*, 12126-12133, doi:10.1021/jp012542o.
2. Ciccotti, G.; Ferrario, M.; Ryckaert, J.P. Molecular dynamics of rigid systems in cartesian coordinates A general formulation. *Molecular Physics* **1982**, *47*, 1253-1264, doi:10.1080/00268978200100942.
3. Tanis, I.; Brown, D.; Neyertz, S.; Heck, R.; Mercier, R.; Vaidya, M.; Ballaguet, J.-P. A comparison of pure and mixed-gas permeation of nitrogen and methane in 6FDA-based polyimides as studied by molecular dynamics simulations. *Computational Materials Science* **2018**, *141*, 243-253, doi:https://doi.org/10.1016/j.commatsci.2017.09.028.
4. Yin, D.; MacKerell, A.D. Combined ab initio/empirical approach for optimization of Lennard–Jones parameters. *Journal of Computational Chemistry* **1998**, *19*, 334-348, doi:10.1002/(sici)1096-987x(199802)19:3<334::aid-jcc7>3.0.co;2-u.
5. Humphrey, W., Dalke, A. and Schulten, K., VMD - Visual Molecular Dynamics, *J. Molec.* Graphics **1996**, *14*, 1, 33-38.

Disclaimer/Publisher's Note: The statements, opinions and data contained in all publications are solely those of the individual author(s) and contributor(s) and not of MDPI and/or the editor(s). MDPI and/or the editor(s) disclaim responsibility for any injury to people or property resulting from any ideas, methods, instructions or products referred to in the content.
